# Atlanta Society of Medicine

**Published:** 1890-11

**Authors:** 


### ATLANTA SOCIETY OF MEDICINE.

---

Meeting, October 7th. President Dr. Nicolson in the chair.

ECZEMA PAPULATUM VENENATUM.

Dr. Virgil O. Hardon reported a case of

MYOMOTOMY IN A PREGNANT WOMAN.

The patient, a primipara, four months advanced in pregnancy, noticed about two months ago a lump in the right side which rapidly increased in size and gave rise to severe pain, especially upon walking. For the last two weeks the pains simulated those of labor, and she feared that a miscarriage would result; in other respects her health was good.

Examination revealed a round tumor in the right inguinal region, freely movable, easily felt through the abdominal wall, and as large as a good-sized orange. The uterus was pushed over to the left side. By vaginal examination the tumor could be easily felt and was apparently not connected with the uterus. It was solid and had the feeling of a fibroma. Its removal was recommended.

On the 25th of September, with the assistance of Drs. Harris and Kennedy, the abdomen was opened in the median line. The tumor was found to be a uterine fibroid connected with the uterus by a pedicle as large as one's wrist. It was drawn out through the incision and the pedicle ligated in three portions and divided by scissors. The stump was dropped back into the abdomen. The incision was closed without drainage. The patient made a rapid and uneventful recovery, and her pregnancy is now progressing without interruption.

Dr. Hutchins reported a case which should be of interest. Last summer a young gentleman was referred to him by Dr. Jones. The skin eruption had been present three weeks, and was limited to ankles, around, and about three inches above and below. Consisted of papules which were very "itchy," "burning" when scratched. No evidence of vesiculation. Tops of papules were slightly crusted—result of scratching. Eruption thought to have been produced by dye in a certain kind of black socks worn.

Recovered in about ten days, under washing with "spir. sapon-kalinus," and the use of oxide of zinc ointment containing a drachm of liquid pitch to the ounce. A few days ago the patient reported that while North in the summer he wore the same socks, and, with high shoes, the eruption recurred, but did not with low shoes. Evidence that the dye produced the eruption is good. Its occurrence only when high shoes were worn would seem to indicate that confinement of secretions was necessary to its production, furthering absorption through the hair follicles. The papular character of the eruption and the fact that few hairs grow below the lower border would sustain the view of follicular absorption and explain non-occurrence on the feet. Substitution of white socks relieved symptoms.

Lapartomy.—Dr. Earnest reported an abdominal section. Patient age thirty-two, married several years, has never been pregnant. Sought advice on account of pain in ovarian region and excessive and painful menstruation. The pain of late at the period has been excruciating, and usually accompanied with vomiting the first twenty-four hours. The uterus was retroverted, and lying close up against it on the left side was felt a tumor the size of a large orange. The whole mass was glued together and immovable, forming a dense, stiff vaginal vault. October 1st, assisted by Drs. Jarnigan and Elkin, the abdomen was opened and a cyst the size of an orange was found firmly imbedded in Douglas' pouch, and nearly covered in by the overlapping uterus and a fold of broad ligament. With some difficulty it was carefully dug out and brought into view. The broad pedicle was tied with silk and the tumor cut away. A knotty fibroid the size of a guinea egg was then removed from the fundus uteri. The wound was closed with silk. Strict antisepsis was observed throughout. The patient has done well.

Dr. Jones reported following case:

September 19th, 11 p. m.—Called to see a negro woman said to be in labor; on arriving found a white midwife who told me that the patient had been vomiting for several hours and that her womb was turned upside down and she could not do anything with it. Made examination and found uterus in proper position, but labor not set in. Ordered a dose of castor oil and left. Was sent for again on 21st, about 6 p. m., and found the woman in labor; on making examination found the os dilated as large as a silver dollar, and the sack of water pressed firmly down into it. On waiting for an intermission of pain to make further examination and diagnosis of presentation, I discovered that there was no relaxation, but that the uterine contraction was continuous. On inquiry learned that the midwife in attendance had give two or three doses of fluid extract of ergot during the afternoon. About this time the membranes ruptured and I found on examination a breech presentation. The uterine contraction remaining continuous and increasing in force, the woman was speedily delivered of a living female child. Without intermission the uterine contraction continued, and in about ten minutes a dead male child was born, the breech likewise presenting. The children being small, the patient was delivered rapidly and without injury to the soft parts. The placenta was expressed, the uterus remained contracted and the patient convalesced rapidly.

---

### ATLANTA SOCIETY OF MEDICINE.

Regular meeting, October 21st. President Dr. Nicolson in the chair.

### THE TREATMENT OF PERITONITIS.

Dr. Baird said, in opening the discussion of the treatment of peritonitis, he experienced some embarrassment in the attempt to present this branch of the subject, entirely divorced from considerations of causation of the several varieties of the disease, and of the usual stages which make its career, but he would confine himself to a review of the medical treatment only of acute diffuse or general peritonitis, as usually encountered after development, by the physician at the bedside, and he would also endeavor to condense his remarks and to observe the limit of ten minutes allowed him.

The plan of treatment which has been followed for the last quarter of a century, or more, by the great majority of the American physicians, is commonly known as the "opium treatment," and it is still generally approved and practiced.

This plan consists in the early and free administration of opium until the patient shall have been brought to a state of semi-narcotism that is, to a point where the pain is relieved; the respirations are reduced to twelve or fourteen per minute, and the sleep is sound. In this state we may expect improvement in the pulse and in the tympanitis. This point being reached, it should be cautiously and watchfully maintained. Care should be taken not to go too far or to push the administration of the drug to the degree of complete or profound narcotism, in which it is difficult to arouse the patient, or in which the respirations fall below the limit already suggested. A remarkable tolerance of opium in this disease is sometimes manifested, and what would seem excessive doses may be required in order to procure the desired result. It is then necesary for the physician to personally supervise its exhibition, and to determine the requisite amount to be used, by noting attentively the effects produced. It is asserted by the critics of this mode of treatment that it "masks the symptoms" of the disease, and is thus calculated to mislead the attendant.

Pain is a distressing symptom, and we do not object to its being masked or indeed entirely obliterated. Rapid respiration is a symptom which we need not seek to perpetuate. The wiry pulse and the tympanitis need not be preserved in their menacing integrity for our guidance. The subjective symptoms may be dispersed with somnolence and ease may be allowed the patient, so long as we have the finger boards furnished by the pulse, the respiration and the degree of the abdominal distention to guide us. The form of opium employed may be selected to suit best the exigencies of the case. Morphine, powdered opium or the liquid preparations may be used either hypodermically, by the mouth or by the rectum. It is important to secure the desired effect early and to maintain it uninterruptedly and uniformly.

The treatment by cathartics, recently advocated as a new method, is not new, but is a return to an old and discarded treatment. Saline cathartics are especially lauded, and when they are not tolerated by the stomach, calomel is substituted; not for any supposed sorbefacient or antiplastic action, but for its cathartic effect alone. It seemed to him that if any indications for cathartics exist, it must be in the very first stage of the affection, before the disease is fully developed, and usually before the patient is seen by the physician. After full development, the indications for treatment are best met by quietude of the body, by quietude of the inflamed structure, and by effort to control unfavorable symptoms as they arise. Conservatism has its place in medical practice as well as in surgery. It does not contravene active treatment, but it does demand circumspection and good judgment in the application of remedies. General blood-letting need scarcely be referred to. It was ranked among the antiphlogistic remedies, along with drastic purgatives of a former age. Local bleeding by means of leeches to the abdomen is yet approved by some able men, in the outset of the disease. Of the topical applications, the milder counter-irritants are to be recommended. Sinapism, turpentine frictions, or stupes and warm fomentations may be advantageously employed. Turpentine injections and, possibly, salol may be serviceable in overcoming tympanitis. Blisters are not advisable. They rank among the perturbing measures that should be avoided. Cold applications, by means of the ice water coils, clothes dipped in ice water or ice bags, are preferred to the warm by some practitioners. Puncture of the intestines, in cases of extreme distention, may be regarded as a medical operation, but he only referred to it for the purpose of saying that, in his opinion, it was not free from danger. Antipyretics may be indicated in case of hyperpyrexia. Obstinate vomiting may require palliative treatment by means of ice, champagne, etc., and the tendency toward asthenia should be combatted by judicious alimentation, by stimulants, and by sustaining measures.

Dr. Gaston said, while he was expected to deal with the subject from a surgical standpoint, he did not consider himself confined to simply a discussion of operative measures. He thought puncture o the bowel unsurgical and unsafe. He would employ external applications, such as turpentine, camphor, mustard, warm fomentations, etc. He would not operate except in suppurative peritonitis.

Dr. Virgil O. Hardon said :

The treatment of peritonitis by large doses of opium has been practiced for more than a quarter of a century. The results have been only moderately good. Peritonitis has during that time been regarded as a dangerous and often fatal disease. A few years ago, Tait startled the medical world by announcing the astonishing results which he had obtained in treating this disease by means of saline laxatives. So great an innovation upon established practice was slow in meeting with acceptance at the hands of the profession, and even at the present day this method of treatment is almost confined to specialists in gynecology. But to those who have had an opportunity to observe the effect of this treatment, the results obtained come like a revelation. Instead of a dangerous, tedious, protracted period of ten days or two weeks of suffering, the disease is immediately cut short and the patient goes on to a rapid convalescence. Take, for instance, a well marked case of peritonitis after laparotomy, with the temperature at 104, the pulse 130, the abdomen tympanitic and the patient in extreme pain. If Epsom salts be given in teaspoonful doses every hour in hot water until watery stools are induced, in from twelve to twenty-four hours the temperature will have fallen below 100, the pulse to 80, the tympanites will have disappeared, the pain will have been as thoroughly relieved as it would be by morphine and the patient will be convalescent These results are so constant that Epsom salts may be regarded as a specific in peritonitis as much as quinine is in intermittent fever or mercury in syphilis. The consequence is that peritonitis is no longer a disease to be feared as it formerly was. Under the opium treatment a large percentage of the deaths after abdominal operations were from this cause. It is now a rare thing to hear of a death from peritonitis after operation.

The same is true of peritonitis, starting in the pelvis from disease of the tubes or ovaries or following labor. The same treatment by Epsom salts will produce equally good results in these cases. It will effectually abort the disease, and the patient will be convalescent within twenty-four hours. There are women with diseased Fallopian tubes who are subject to recurrent attacks of pelvic peritonitis and who, under any other method of treatment, would be sick for ten days or a fortnight at each attack, followed by a protracted convalescence. I have had a number of such patients who have learned that their periodical attacks can be effectually aborted by Epsom salts, and who successfully treat themselves in this way without calling in a doctor. One such patient remarked to me that "since she had learned to use Epsom salts she would rather have an attack of pelvic peritonitis than to have a bad cold."

The results obtainable by this plan of treatment are so immeasurably superior to those yielded by the opium treatment, that I have never seen a man who had had an opportunity to compare the two who did not give the preference to the new method. The reason it is not more universally in vogue with the profession at large, is because its superiority is not generally known. It is so diametrically opposed to the idea of "putting a splint on the bowels," which has been taught for the last quarter of a century, that it is difficult to break down the prejudice which has been engendered thereby. The objections to this method are all purely theoretical, and are fully met by clinical results. There are gentlemen here to-night who have, at my suggestion, tried this plan of treatment. They did so with reluctance and many misgivings. They were astonished and delighted at the results obtained, and are to-day enthusiastic in its praise.

Dr Todd said he had met with comparatively few cases of general peritonitis, and he was inclined to consider the disease rather infrequent. He had generally used some form of opium, preferably morphine, hypodermatically. He did not agree with Dr. Baird as to the administration of gum opium per orum, on account of its slow absorption, and the danger of surcharging the system. He preferred that the bowels should act well. Counter-irritation he esteemed valuable, especially a cantharidal blister. He was of the opinion that the treatment of peritonitis by saline cathartics was the best.

Dr. Cooper said he differed with Dr. Baird as to the best treatment. His experience with the opium treatment had been very unsatisfactory to him, and disastrous to his patients. To him the idea of a blister in acute general peritonitis was simply horrible. He mentioned three cases of peritonitis treated with salines with the happiest results. He thought in the light of our present experience that opium should be discarded in the treatment of peritonitis and that we should rely on salines.

Dr. Howell said he was brought up in the faith of the opium treatment and that almost all of the cases which he had seen treated with opium had died. Recently he had treated a young man suffering with a very severe peritonitis with opium for several days without amelioration of the symptoms. As a last resort, upon the advice of Dr. Hardon, he put him on the saline treatment with the happiest results, the relief being almost immediate and permanent.

Dr. McRae said, that owing to his having been absent from the city he was not aware what the subject for discussion was until he came to the meeting. He had, however, heard the same subject very thoroughly discussed by the members of the New York County Medical Society. The same differences of opinion were expressed there as here, and while the majority favored the saline treatment as a rule, there seemed to be a general consenus of opinion in favor of opium in selected cases. Several of the gentlemen preferred codeia to morphine or any of the other preparations of opium. Personally he had had comparatively a limited experience with peritonitis. He had only seen three cases, all of which were of pelvic origin and were treated with salines. The results had been very satisfactory.

Dr. Elkin said he believed a localized peritonitis almost without exception preceded general peritonitis. He would rely on saline rather than opiates.

Dr. Huzza reported three cases of puerperal peritonitis, all in negroes, under the most unfavorahle hygienic surroundings that had abated promptly upon thoroughly purging the patients with cathartics other than salines. He expressed himself as decidedly in favor of the saline treatment.

In closing the debate, Dr. Baird said that he was not a partisan, and that he was willing always to exchange any practice for another proven superior, and to relinquish any views which were not sustained by the test of experience. However, when he heard the enthusiastic eulogies often heaped upon new remedies or the new application of old remedies, he is reminded of a crafty old French cardinal, whose habit it was to piece out the lion's skin, where it fell short, by adding the fox's. As a high military officer left his presence, with a very profound obeisance, he shrewdly observed: "I half suspect that fellow, he bows too low." So, his confidence is sometimes shaken when gentlemen claim too much. He should be glad to know that in Epsom salts we had, as we might infer from what we have just heard, an infallible specific for this formidable disease, and not only this, but also an infallible preventive, if we accept the evidence of some recent writers upon this subject. This disease would thus be practically annihilated. The remarks of Dr. McRae foreshadowed the development of a more conservative estimate of the power of Epsom salts. It is not destined to displace opium, however helpful it may be in certain cases or in certain stages of the disease.

Its prophylactic power, if it possessed such, is scarcely available to the physician in general practice. The question of diagnosis is an important element in estimating the value of drugs. Reference has been made to the importance of forcing a prompt absorption of the exudation as affording an indication for purgatives. It should be remembered that this exudation is the product, not the cause, of the inflammatory condition. The serum is harmless, and can be easily managed after the inflammation shall have subsided. Nobody expects to secure absorption of the fibrin, or of the pus when present in considerable quantities. Death may result from shock or from slow or rapid asthenia. Opium is not only palliative, but is curative. It renders the system tolerant of the disease. It is probable that in the suppurative variety there exists a specifiic infective cause; possibly a micro-organism, and it is more than likely that the traumatic, and the so-called idiopathic forms of the disease are due to different etiological factors, and not present identical pathological conditions. Traumatic and so-called spontaneous pleurisy and traumatic and epidemic meningitis hold some sustaining relation to this line of thought. The disease, as encountered in connection with abdominal operations, and the disease met with by the physician in general and in obstetric practice, is not necessarily one and the same in every respect.

